# Mitral Transcatheter Edge-to-Edge Repair In-Hospital Outcomes and Mitral Valve Surgery Readmission Trends: National Readmission Database 2018-2020

**DOI:** 10.1016/j.shj.2024.100350

**Published:** 2024-07-27

**Authors:** Abdul Rahman Akkawi, Syed Zaid, Taha Hatab, Rody G. Bou Chaaya, Emmanuel Oundo, Nadeen Faza, Stephen H. Little, Marvin D. Atkins, Michael J. Reardon, William A. Zoghbi, Neal S. Kleiman, Sachin S. Goel

**Affiliations:** a Department of Internal Medicine, University of Kansas School of Medicine, Wichita, Kansas; b Department of Cardiology, College of Medicine, Michael E. DeBakey VA Medical Center, Houston, Texas; c Department of Cardiology, Houston Methodist DeBakey Heart and Vascular Center, Houston, Texas

**Keywords:** Mitral valve regurgitation, Mitral valve replacement, Mitral valve surgery, Transcatheter edge-to-edge repair

## Abstract

• Study design: Utilized the National Readmission Database from 2018-2020. The study included patients over 18 years of age who underwent transcatheter edge-to-edge repair and analyzed readmission for mitral valve (MV) surgery within 180 days.
• Readmission rate: Only 1.1% of patients who underwent transcatheter edge-to-edge repair required MV surgery within 180 days.
• Mortality and morbidity: In-hospital mortality after MV surgery was 9.7%, with a high incidence of acute kidney injury (51%) and bleeding events (15.7%) among readmitted patients.
• Trend over time: The readmission rate for MV surgery significantly declined from 1.8% in 2018 to 0.8% in 2020.
• Predictors of readmission: Younger age was identified as an independent predictor of readmission for MV surgery.

## Introduction

Over the past decade, advances in transcatheter interventions have transformed the landscape of treating symptomatic mitral valve (MV) disease. Mitral transcatheter edge-to-edge repair (TEER) is now approved for high-risk surgical symptomatic patients with moderate-to-severe or severe primary mitral regurgitation (MR) and those with moderate-to-severe or severe secondary MR despite optimal medical therapy and favorable MV anatomy.^1^ Despite TEER demonstrating a favorable safety profile and durable MR reduction, some patients still require subsequent MV intervention due to recurrent or residual MR.^2^^,^^3^ We sought to investigate MV surgery readmission rates, in-hospital outcomes, and predictors of readmission following mitral TEER.

## Methods

This is an observational retrospective analysis from the National Readmission Database 2018-2020, which is derived from the Healthcare Cost and Utilization Project. The National Readmission Database includes discharges for patients with and without repeat hospital visits and those who died in-hospital. The database’s standardized sampling and weighting method provided by the Agency for Healthcare Research and Quality enabled national estimates for the entire US hospitalized population.^4^

Our study included all patients over 18 years of age undergoing mitral TEER who were discharged alive and had a subsequent readmission within 180 days after discharge. We examined the index TEER admission and the subsequent readmission for MV surgery, analyzing a range of factors including patient demographics, comorbidities, and hospital characteristics. The primary outcome was readmission for MV surgery within 180 days post-TEER, which included both MV replacement (MVR) and MV repair (MVr). A logistic regression model calculated adjusted odds ratios with 95% CIs for predictors for MV surgery readmission. The multivariable logistic regression model adjusted for potential factors including patient demographics, comorbidities, and hospital characteristics. We also analyzed the trend of readmission for MV surgery.

## Results

**Figure 1:**
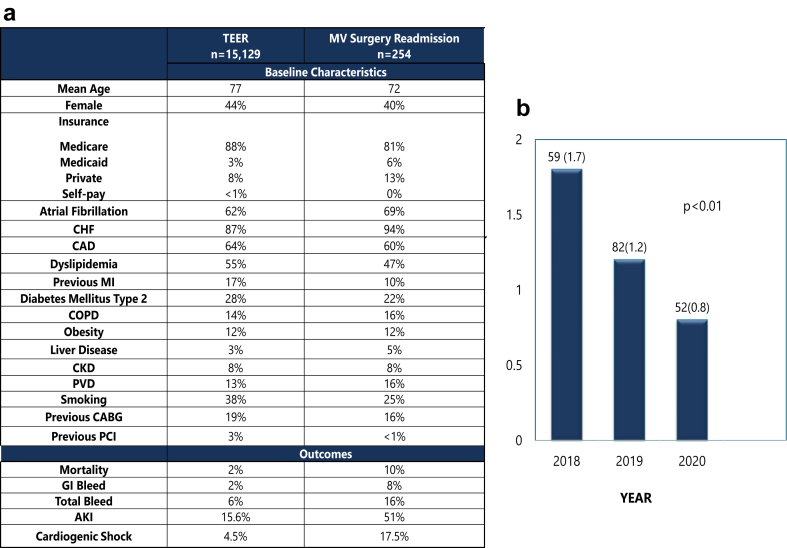
(a) The baseline characteristics and outcomes of principle TEER admission and MV surgery readmission. (b) MV surgery trend post-TEER between 2018 and 2020 Abbreviations: AKI, acute kidney injury; CABG, coronary artery bypass grafting; CAD, coronary artery disease; CHF, congestive heart failure; CKD, chronic kidney disease; COPD, chronic obstructive pulmonary disease; GI, gastrointestinal; MI, myocardial infarction; MV, mitral valve; PCI, percutaneous coronary intervention; PVD, peripheral vascular disease; TEER, transcatheter edge-to-edge repair.

A total of 15,129 TEER procedures were performed during the study period. Mean age was 77.1 years, and 44% were women (Figure 1). During the index admission, 15.6% had acute kidney injury (AKI), and 5.6% suffered from bleeding events (intracranial hemorrhage, gastrointestinal bleeding, hemoptysis, hematuria, epistaxis, and unspecified bleed) (Figure 1).

The readmission rate for MV surgery at 180 days was 1.1%. Mean age of readmitted patients was 71.8 years, with women representing 39% of this subgroup. The vast majority of those readmitted for MV surgery had MVR (95%), and only 5% underwent MVr. The in-hospital mortality after MV surgery was 9.7%. AKI was reported in 51% of patients, and 15.7% had a bleeding event. Younger age was an independent predictor of readmission for MV surgery (adjusted odds ratio: 0.97 per year, 95% CI 0.95-0.98, *p* ​< ​0.01). The adjusted trend analysis for 180-day readmission for MV surgery during the study period showed a significant decline from 1.8% in 2018 to 0.8% in 2020 (*p* ​< ​0.001) (Figure 1).

## Discussion

Using the national database of patients undergoing MV surgery after TEER, our study sheds light on the significant morbidity and mortality of MV surgery following mitral TEER and provides further insight on the associated temporal trends. Our key findings are as follows: First, MV surgery after mitral TEER is rare, with 1.1% of patients undergoing TEER requiring MV surgery within 6 months of the index procedure. Second, in-hospital mortality after MV surgery for failed TEER is significant; AKI and bleeding events were more common in the MV surgery group compared to the index mitral TEER admission. Third, there was a downward trend in MV surgery rates after mitral TEER during the study period (2018-2020). Finally, advanced age was associated with a decreased likelihood of undergoing MV surgery within 180 days.

The in-hospital mortality rate of 9.7% among patients readmitted for MV surgery aligns with existing literature. A study by Kaneko et al. from the CUTTING-EDGE registry reported 15.1% in-hospital mortality following MV surgery for failed TEER.^1^ This observation underscores the clinical complexity associated with MV surgery in this subset of high-risk surgical patients. Our study also showed that less than 10% of patients who had MV surgery underwent MVr following failed TEER, a trend consistent with previous reports from registries such as the CUTTING-EDGE registry and reports from the Society of Thoracic Surgeons database.^1^^,^^5^ The low repair rate is expected because MVR usually provides a more direct and reliable outcome, leaving no residual MR in patients with higher surgical risks.^1^ Also, TEER might make MVr less feasible during follow-up procedures.^1^

The AKI rates for index TEER and MV surgery were 15.6% and 51%, respectively. These findings align with existing literature, as Taramasso's research revealed that approximately 23.8% of patients developed AKI following the MitraClip procedure.^6^ Similarly, Chang et al.^7^ found that 38.7% of patients who underwent MV surgery experienced AKI, which was associated with adverse outcomes and higher mortality rates. One explanation is that patients undergoing MV surgery have more comorbidities affecting kidney function; additionally, patients with MR often have decreased stroke volume, increasing the risk of renal injury during cardiac procedures. Furthermore, the declining trend in 180-day readmissions for MV surgery over the study years prompts reflection on potential underlying factors driving this pattern. One plausible explanation could be an evolving approach among clinicians toward more optimizing patient selection for index TEER procedure and subsequent MV surgery after failed TEER. Although patients with failed TEER who were declined surgery were not captured in the registry, the declining trend in readmission for MV surgery may reflect enhanced risk assessment protocols aimed at identifying patients most likely to benefit from additional surgical interventions while minimizing associated risks. Alternatively, improvements in transcatheter techniques, newer device generations, operator experience, and postoperative care protocols may have contributed to better outcomes following TEER procedures, thereby reducing the necessity for repeated MV surgeries.

Our study has certain limitations. Our analysis is descriptive in nature, and we did not conduct regression analysis to identify factors associated with in-hospital mortality after MV surgery as we were limited by the number of events. Moreover, while it is crucial to determine the etiology of MR that led to TEER, our study is limited by the lack of detailed baseline and procedural characteristics, such as cause of MR, echocardiographic data, and indication for TEER; this prevents a comprehensive analysis of whether the MR was primary or secondary. Additionally, as with any retrospective study utilizing Healthcare Cost and Utilization Project data, our analysis is subject to inherent limitations such as potential coding errors, missing echocardiographic and procedural data, and the inability to establish causality.

## Conclusions

While MV surgery after TEER is rare, it is associated with significant in-hospital mortality and morbidity. As transcatheter therapies continue to evolve, detailing readmission trends and complications in TEER patients becomes crucial, as it may impact the lifetime management of patients with MR. Future research is warranted to explore factors influencing suboptimal outcomes after TEER, with a focus on mitigating risks associated with subsequent surgical interventions.

## Ethics Statement

Ethical review and approval were waived for this study because the dataset is deidentified, publicly available, and does not involve the use of test materials.

## Funding

The authors have no funding to report.

## Disclosure Statement

M. J. Reardon is a consultant for Medtronic, Boston Scientific, Abbott, and W. L. Gore & Associates. M. D. Atkins is a consultant for W. L. Gore & Associates. N. S. Kleiman is a local principal investigator in trials sponsored by Boston Scientific, Medtronic, Abbott, and Edwards Lifesciences. S. S. Goel is a consultant for Medtronic, W. L. Gore & Associates, and on the speakers bureau for Abbott Structural Heart. The other authors had no conflicts to declare.
